# The generation of stable transgenic lines in the human-infective nematode *Strongyloides stercoralis*

**DOI:** 10.1093/g3journal/jkae122

**Published:** 2024-06-05

**Authors:** Ruhi Patel, Astra S Bryant, Michelle L Castelletto, Breanna Walsh, Damia Akimori, Elissa A Hallem

**Affiliations:** Department of Microbiology, Immunology, and Molecular Genetics, University of California, Los Angeles, Los Angeles, CA 90095, USA; Department of Physiology and Biophysics, University of Washington, Seattle, WA 98195, USA; Department of Microbiology, Immunology, and Molecular Genetics, University of California, Los Angeles, Los Angeles, CA 90095, USA; Department of Microbiology, Immunology, and Molecular Genetics, University of California, Los Angeles, Los Angeles, CA 90095, USA; Molecular Biology Interdepartmental PhD Program, University of California, Los Angeles, Los Angeles, CA 90095, USA; Medical Scientist Training Program, University of California, Los Angeles, Los Angeles, CA 90095, USA; Department of Microbiology, Immunology, and Molecular Genetics, University of California, Los Angeles, Los Angeles, CA 90095, USA; Molecular Biology Interdepartmental PhD Program, University of California, Los Angeles, Los Angeles, CA 90095, USA; Department of Microbiology, Immunology, and Molecular Genetics, University of California, Los Angeles, Los Angeles, CA 90095, USA; Molecular Biology Institute, University of California, Los Angeles, Los Angeles, CA 90095, USA

**Keywords:** parasitic nematode, *Strongyloides stercoralis*, skin-penetrating nematode, threadworm, parasitic helminth, genetic manipulation, transgenesis

## Abstract

The skin-penetrating gastrointestinal parasitic nematode *Strongyloides stercoralis* causes strongyloidiasis, which is a neglected tropical disease that is associated with severe chronic illness and fatalities. Unlike other human-infective nematodes, *S. stercoralis* cycles through a single free-living generation and thus serves as a genetically tractable model organism for understanding the mechanisms that enable parasitism. Techniques such as CRISPR/Cas9-mediated mutagenesis and transgenesis are now routinely performed in *S. stercoralis* by introducing exogenous DNA into free-living adults and then screening their F_1_ progeny for transgenic or mutant larvae. However, transgenesis in *S. stercoralis* has been severely hindered by the inability to establish stable transgenic lines that can be propagated for multiple generations through a host; to date, studies of transgenic *S. stercoralis* have been limited to heterogeneous populations of transgenic F_1_ larvae. Here, we develop an efficient pipeline for the generation of stable transgenic lines in *S. stercoralis.* We also show that this approach can be used to efficiently generate stable transgenic lines in the rat-infective nematode *Strongyloides ratti*. The ability to generate stable transgenic lines circumvents the limitations of working with heterogeneous F_1_ populations, such as variable transgene expression and the inability to generate transgenics of all life stages. Our transgenesis approach will enable novel lines of inquiry into parasite biology, such as transgene-based comparisons between free-living and parasitic generations.

## Graphical Abstract

**Graphical Abstract:**
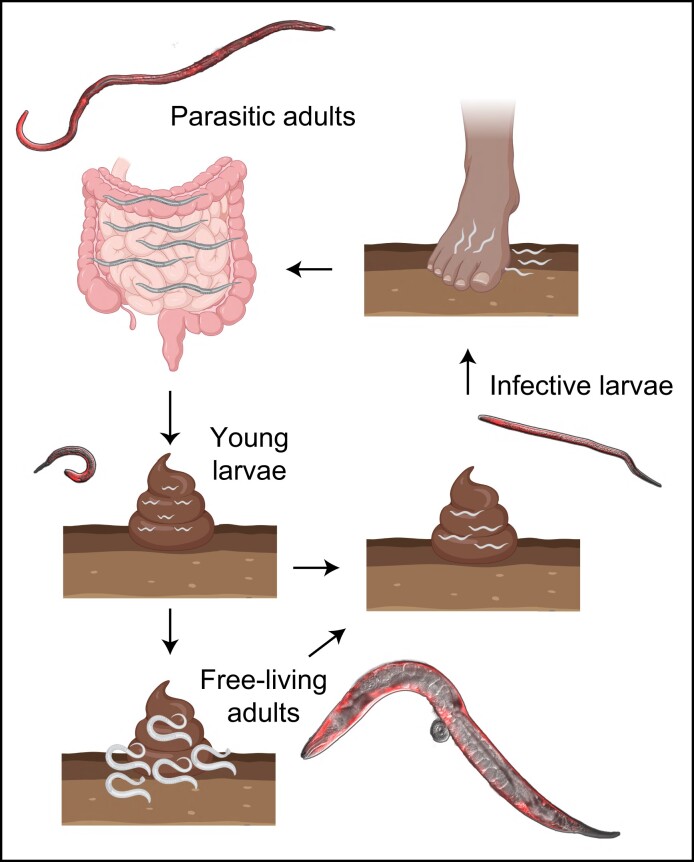

## Introduction

Soil-transmitted helminths cause some of the most prevalent human infections, with current estimates suggesting that ∼2 billion individuals are infected worldwide (Riaz *et al.* 2020; WHO 2023). The skin-penetrating parasitic nematode *Strongyloides stercoralis* is a soil-transmitted helminth that infects ∼610 million people globally (Buonfrate *et al.* 2020) and disproportionately affects low-resource communities that lack access to basic necessities such as proper sanitation infrastructure (Beknazarova *et al.* 2016). Symptoms of *S. stercoralis* infection include gastrointestinal and respiratory distress, diarrhea, and vomiting (Kassalik and Monkemuller 2011; Czeresnia and Weiss 2022). Notably, *S. stercoralis* infections in immunocompromised individuals can progress to disseminated strongyloidiasis and hyperinfection syndrome, of which most cases result in death due to conditions such as respiratory failure and meningitis (Kassalik and Monkemuller 2011; Buonfrate *et al.* 2013; Czeresnia and Weiss 2022). Resistance to the drug ivermectin, the primary treatment for strongyloidiasis, has been reported among *Strongyloides* species that infect livestock (Maroto *et al.* 2011), forewarning the possibility of anthelmintic resistance in *S. stercoralis* populations. Together, the morbidity and socioeconomic burden caused by *Strongyloides* infections and the threat of resistance to existing treatments necessitate the development of preventative measures and new therapies.

**Fig. 1.**
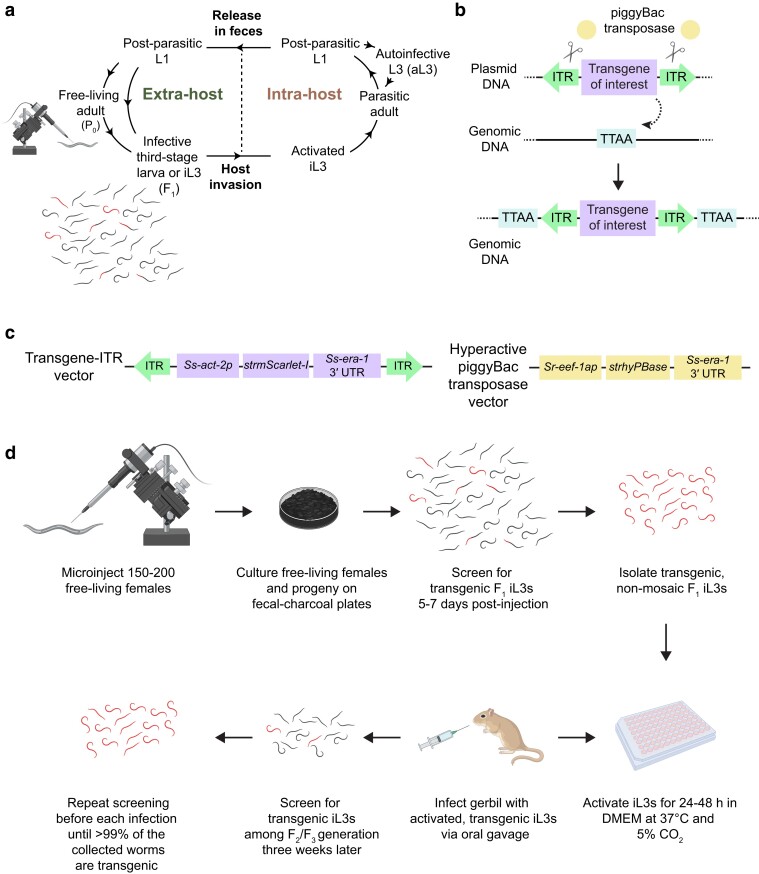
A framework for high-efficiency transposase-mediated integration of transgenes in *S. stercoralis*. a) The life cycle of *S. stercoralis* is composed of stages that live within the host (intra-host) and outside the host (extra-host). Developmentally arrested iL3s invade the host body by penetrating through the skin. Development resumes in a process called activation, and activated iL3s then progress through multiple life stages to become reproductively mature parasitic adults. Parasitic adults live and lay eggs inside the host duodenal mucosa. The eggs hatch and release post-parasitic L1s, which either exit the host in feces or develop into aL3s that reinfect the host. Outside the host, post-parasitic L1s may develop into iL3s or free-living adults; the progeny of free-living adults develop exclusively into iL3s. The gonads of free-living adults (*i.e.*, the P_0_ generation) are routinely microinjected with DNA to generate transgenic F_1_ iL3s that express the transgene from extrachromosomal arrays. However, there are 3 main issues associated with working with extrachromosomal arrays: (1) transgenic iL3s, which are depicted in color, constitute only a small fraction of the total progeny; (2) transgenes might show mosaic expression, as represented by the partially colored worms; and (3) extrachromosomal arrays are silenced from the F_2_ generation onward, impeding use of these arrays for studies of post-parasitic larvae, aL3s, and free-living adults. b) The mechanism of action of the piggyBac transposase system. The piggyBac transposase precisely excises fragments of DNA that are flanked by ITRs and inserts them into 5′-TTAA-3′ motifs in the genome, duplicating the 5′-TTAA-3′ motif upon insertion. c) Plasmid vectors used for hyPBase-driven chromosomal integration. The transgene–ITR vector comprises the transgene to be integrated, flanked by ITRs. The transgene depicted here contains a promoter fragment of the *S. stercoralis act-2* gene fused to a gene encoding the fluorescent protein mScarlet-I and the 3′ UTR of the *S. stercoralis era-1* gene. The hyPBase expression vector contains a *Strongyloides*-codon–optimized gene encoding hyPBase, flanked by the promoter of the ubiquitously expressed *S. ratti eef-1a* gene and the 3′ UTR of *Ss-era-1*. d) Strategy for generating a stable transgenic line in *S. stercoralis*. Between 150 and 200 free-living females are microinjected with a DNA mixture composed of the hyPBase expression vector and the transgene–ITR vector of interest. The free-living females are recovered and allowed to lay progeny on fecal–charcoal plates. After a minimum of 5 days, fluorescence microscopy–based screening is performed to isolate transgenic F_1_ iL3s that show transgene expression in all the expected cell types (depicted here as fully colored worms). The iL3s that show mosaic expression (depicted as partially colored worms) are excluded. Isolated iL3s are activated by incubation in conditions that mimic the intra-host environment. Activated transgenic iL3s are propagated by inoculation into a single gerbil via oral gavage. Three weeks later, the F_2_/F_3_ iL3s are collected from coprocultures made with feces from the infected gerbil; the F_2_/F_3_ iL3s are screened for transgene expression. If transgenic iL3s are detected, they are isolated and used for the next round of infections. Screening is repeated prior to each round of the infection until >99% of the worms collected from the gerbils are transgenic.

The life cycle of *S. stercoralis* is complex and composed of life stages that reside in both extra-host and intra-host environments (Fig. 1a; Viney and Lok 2015; Buonfrate *et al.* 2023). Parasitic adults inhabit the host duodenal mucosa and reproduce to yield post-parasitic first-stage larvae (L1s). Post-parasitic L1s may develop directly into autoinfective third-stage larvae (aL3s) within the host body, which then reinfect the host. Alternatively, post-parasitic L1s may be expelled from the host, in feces, into the environment. Outside of the host, the larvae follow one of two developmental routes. In the first route, they develop directly into the infective third-larval stage (iL3). In the second route, the larvae instead develop into free-living adults, which mate and reproduce on host feces to produce progeny that develop exclusively into iL3s. The iL3s are developmentally arrested and nonfeeding, like the dauer larvae of the free-living nematode *Caenorhabditis elegans* (Viney *et al.* 2005; Crook 2014), and must infect a host to continue their life cycle. When iL3s encounter a suitable host, they invade the body by penetrating through the skin. Inside the host, iL3s transition to activated iL3s (i.e. they start to feed and resume development) and ultimately become parasitic adults that colonize the duodenum (Dionisio *et al.* 2000; Viney and Lok 2015; Buonfrate *et al.* 2023).

*S. stercoralis* is a genetic model system for the study of human-infective nematodes because its ability to cycle through a free-living generation makes it uniquely amenable to genetic manipulation (Castelletto *et al.* 2020). *S. stercoralis* free-living adults are similar in size and morphology to *C. elegans* adults (Altun and Hall 2009; Castelletto *et al.* 2023). Notably, both nematode species have syncytial gonads, and thus, techniques that were developed in *C. elegans* for delivery of transgenes and other cargo via intragonadal microinjection have been successfully adapted to *S. stercoralis* (Lok 2007; Castelletto and Hallem 2021). As a result, several genetic techniques for probing the biology of *S. stercoralis* have already been developed (Castelletto *et al.* 2020; Mendez *et al.* 2022). Visualization of gene expression patterns, cell morphology, and neuronal activity has been achieved by intragonadal microinjection of genes encoding transcriptional reporters or biosensors, followed by fluorescence microscopy–based examination of transgenic F_1_s (Lok and Massey 2002; Li *et al.* 2006; Junio *et al.* 2008; Bryant *et al.* 2018, 2022). Chemogenetic silencing of specific neurons to examine the ensuing effect on behavior has been successfully applied in *S. stercoralis* by expressing the histamine-gated chloride channel HisCl1 in neurons of interest followed by treatment of transgenic worms with exogenous histamine (Bryant *et al.* 2022). Genetic inactivation has also been achieved in *S. stercoralis* using CRISPR/Cas9-mediated targeted mutagenesis, where CRISPR components were microinjected into free-living adults and mutant F_1_ larvae were obtained (Gang *et al.* 2017, 2020; Lok *et al.* 2017; Bryant *et al.* 2018; Cheong *et al.* 2021; Wang *et al.* 2021).

One major limitation of transgenesis in *S. stercoralis* is that there was previously no practical approach for generating stable lines for continuous propagation of transgenic worms. Transgenic nematodes generated by microinjection of exogenous DNA usually carry the transgenes in extrachromosomal arrays (Evans 2006; Junio *et al.* 2008; Lok 2012); in *S. stercoralis*, these transgenes are only expressed in the F_1_ generation, presumably because of array silencing in subsequent generations (Junio *et al.* 2008). As a result, transgene-based studies in *S. stercoralis* can only be done with the F_1_ progeny of the microinjected free-living females (P_0_s). Since the F_1_ progeny develop exclusively into iL3s, this limitation precludes the study of certain life stages such as the free-living adult and post-parasitic larval stages (Fig. 1a). When transgenic F_1_s are needed for experiments, these worms must be generated by microinjection of *S. stercoralis* free-living females for each new experimental day; this approach is time-consuming, especially in the case of low rates of transgenesis (Fig. 1a). Additionally, extrachromosomal arrays are associated with mosaic expression of transgenes (Evans 2006; Gang *et al.* 2017), which can be problematic depending upon the experimental goal; for example, when trying to silence a group of neurons using HisCl1 to study the effect on a particular behavior (Pokala *et al.* 2014; Bryant *et al.* 2022), the transgene might be expressed in just a subset of the target neurons, leading to incomplete silencing and variability in the resulting data set. An approach for generating heritable transgenic *S. stercoralis* lines that show consistent transgene expression for multiple generations is necessary to address these issues.

Previous studies have shown that chromosomal integration of transgenes with the piggyBac transposase system in the rat-infective nematode *Strongyloides ratti* bypasses the issues of transgene silencing from the F_2_ generation onward (Lok 2012, 2013; Shao *et al.* 2012). However, this approach produced a transgenesis rate of only 0.03–2.5% in the F_2_ generation (Shao *et al.* 2012). Likewise, the piggyBac transposase system has been used to drive chromosomal integration in other species of parasitic helminths, including the trematode *Schistosoma mansoni* and the filarial nematode *Brugia malayi* (Morales *et al.* 2007; Liu *et al.* 2018). When the piggyBac transposase system was used to drive chromosomal integration in *S. stercoralis*, too few transgenic F_2_ iL3s were recovered for line maintenance (Lok 2012). Maintenance of *S. stercoralis* in the laboratory is normally done by subcutaneous injection of iL3s into gerbils, a laboratory host, with an infective dose of 1,000–2,000 iL3s/gerbil (Nolan *et al.* 1993); however, piggyBac integration in *S. stercoralis* resulted in the generation of only 2 transgenic F_2_ iL3s (0.44% transgenesis rate; Lok 2012).

Here, we present a simple and efficient strategy for generating stable transgenic *S. stercoralis* lines that builds upon the existing technique of using the piggyBac transposase system (Shao *et al.* 2012). In contrast to prior studies, we used the hyperactive mutant version of piggyBac transposase, hyPBase, for genomic integration because hyPBase was shown to have an ∼9-fold increase in integration rates in mammalian cells relative to the wild-type transposase, PBase (Yusa *et al.* 2011). Moreover, the hyPBase that we used was codon-optimized for expression in *Strongyloides* spp. (Bryant and Hallem 2021), unlike the PBase that was used in former studies (Shao *et al.* 2012). Additionally, we activated transgenic F_1_*S. stercoralis* iL3s in vitro (Ashton *et al.* 2007; Stoltzfus *et al.* 2012, 2014; Gang *et al.* 2020) and then infected gerbils via oral gavage with ∼500 activated iL3s. The combination of integration with hyPBase and infection via oral gavage of activated transgenic iL3s resulted in the production of a robust infection with thousands of transgenic F_2_/F_3_ larvae. We have also successfully extended the use of hyPBase-driven chromosomal integration to generate stable lines with *S. ratti*. We anticipate that our approach can be routinely applied to bypass the issues of working with transgenic F_1_s, such as mosaicism and low transgenesis rate, and to enable novel areas of investigation, such as visualizing and comparing neuronal activity or mutant phenotypes across life stages.

## Materials and methods

### Ethics statement

All animal protocols and procedures were approved by the UCLA Office of Animal Research Oversight (Protocol ARC-2011-060). The protocol follows the guidelines set by the AAALAC and the *Guide for the Care and Use of Laboratory Animals*.

### Strains

Wild-type strains used were the *S. stercoralis* UPD strain and the *S. ratti* ED231 strain. The transgenic *S. stercoralis* strain used was EAH435 *bruIs4*[*Ss-act-2p::strmScarlet-I::Ss-era-1* 3′ UTR]. The transgenic *S. ratti* strains used were EAH412 *bruIs1*[*Sr-gcy-23.2p::strYC3.60::Ss-era-1* 3′ UTR], EAH414 *bruIs3*[*Ss-act-2p::strmScarlet-I::Ss-era-1* 3′ UTR], EAH464 *bruIs5*[*Sr-gpa-3p::GFP::Ss-era-1* 3′ UTR], EAH465 *bruIs6*[*Ss-act-2p::mRFPmars:: Ss-era-1* 3′ UTR], and EAH466 *bruIs7*[*Ss-act-2p::mRFPmars:: Ss-era-1* 3′ UTR].

### Maintenance of *S. stercoralis*

*S. stercoralis* was cultured in Mongolian gerbils (Charles River Laboratories) and maintained on fecal–charcoal plates, as previously described (Castelletto *et al.* 2014). To infect gerbils, *S. stercoralis* iL3s were first collected from fecal–charcoal plates using a Baermann apparatus (Lok 2007) and washed 5 times in sterile 1X PBS. After the last wash, the worm pellet was resuspended in 1X PBS at a concentration of ∼10 worms/µL. Each gerbil was anesthetized with isoflurane and inoculated by subcutaneous injection of 200 µL of the worm/PBS suspension, resulting in an infective dose of ∼2,000 iL3s/gerbil; 8–12 gerbils were used for strain maintenance. Feces were collected from days 14 to 44 post-inoculation by housing gerbils overnight on wire racks, over damp cardboard lining (Shepherd Techboard, 8 × 16.5 inches, Newco, 999589), in cages. The next morning, feces were collected from the cardboard using a plastic disposable spoon and then mixed with distilled water (dH_2_O) and autoclaved charcoal granules (bone char, 4-lb. pail, 10 × 28 mesh, Ebonex). Fecal–charcoal mixtures were packed, on top of damp Whatman paper, into 10-cm Petri plates (VWR, 82050-918) and placed in plastic boxes lined with damp paper towels. These boxes were either placed directly in the 23°C incubator or kept at 20°C for 2 days and then moved to a 23°C incubator. *S. stercoralis* free-living females for microinjection were collected from fecal–charcoal plates kept either at 25°C for 1 day or at 20°C for 2 days.

### Maintenance of *S. ratti*

*S. ratti* was cultured in Sprague–Dawley rats (Inotiv) and maintained on fecal–charcoal plates. To infect rats, *S. ratti* iL3s were collected from fecal–charcoal plates using a Baermann apparatus and washed 5 times in sterile 1X PBS. Rats were anesthetized with isoflurane, and each rat was infected, via subcutaneous injection, with ∼700 iL3s in 200 µL of sterile PBS; 2–4 rats were used for strain maintenance. Feces were collected as described above for *S. stercoralis*, except that collections were done from days 7 to 21 post-inoculation. Fecal–charcoal mixtures were made and maintained as described above for *S. stercoralis*. *S. ratti* free-living females used for microinjection were collected either from fecal–charcoal plates kept at 25°C for 1 day or 20°C for 2 days.

### Molecular biology

The plasmid pRP12 (*Ss-act-2p::strmScarlet-I::Ss-era-1* 3′ UTR), which was used for generation of the strains EAH414 and EAH435, contains the promoter of *Ss-act-2*, corresponding to nucleotides 3942973–3944144 of SSTP_scaffold0000001 (*S. stercoralis* genome version PRJEB528-PV0001), except that the sequence 5′-TCTTCTACAGAAACTACACA-3′ in this promoter was replaced with 5′-GCTACCATAGGCACCACGAGCGG-3′. The entire promoter fragment was synthesized by GenScript and then cloned into the vector pMLC201, which contains the *strmScarlet-I* gene (Bryant *et al.* 2022) fused to the *Ss-era-1* 3′ UTR, using the enzymes HindIII (New England Biolabs, R0104) and AgeI (New England Biolabs, R3552). Both pMLC201 and its descendant vector pRP12 have the piggyBac inverted terminal repeats (ITRs) from pPV254 (Shao *et al.* 2012).

To make the hyPBase expression vector pMLC131, the PBase amino acid sequence contained in pPV402 (Shao *et al.* 2012) was first retrieved, and the 7 amino acid substitutions to convert PBase into hyPBase were made manually in Geneious (Geneious Prime 2023.01.1). The codon-optimized coding sequence (CDS) of hyPBase was generated from this amino acid sequence using the Wild Worm Codon Adapter (Bryant and Hallem 2021), and a synthetic intron was added to improve expression of the construct, resulting in the gene sequence of *strhyPBase*. The *strhyPBase* gene was synthesized by GenScript and cloned into pPV540 (Gang *et al.* 2017) using the enzymes AgeI (New England Biolabs, R3552) and AvrII (New England Biolabs, R0174). The resulting plasmid, pMLC131, consists of the promoter of the gene *Sr-eef-1a* (a ubiquitously expressed gene) fused with the *strhyPBase* gene and the 3′ UTR of *Ss-era-1*.

The PBase expression vector pPV402 (*Ss-rps-21p::PBase::Ss-era-1* 3′ UTR) was a gift from Dr. James Lok (University of Pennsylvania). pPV402 consists of the promoter of the gene *Ss-rps-21* (a ubiquitously expressed gene), fused with the CDS of PBase and the 3′ UTR of *Ss-era-1*. The generation of pPV402 was previously described (Shao *et al.* 2012).

The plasmid pASB63 (*Sr-gcy-23.2p::strYC3.60::Ss-era-1* 3′ UTR) was used for generation of the strain EAH412. The strategy for generation of this plasmid was previously described (Bryant *et al.* 2022).

The plasmid pMLC30 (*Sr-gpa-3p::GFP::Ss-era-1* 3′ UTR) was used for generation of the strain EAH464. This plasmid was generated by replacing the *Ss-act-2p* in pPV254 with the promoter of *Sr-gpa-3* from pAJ09 (Addgene, plasmid no. 14913; Junio *et al.* 2008).

The plasmid pMLC24 (*Ss-act-2p::mRFPmars::Ss-era-1* 3′ UTR) was used for making the strains EAH465 and EAH466. pMLC24 was cloned by replacing *GFP* in pPV254 with *mRFPmars* from pAJ50 (Addgene, plasmid no. 14918) using the restriction enzymes HindIII (New England Biolabs, R0104) and AvrII (New England Biolabs, R0174).

Stable lines were also attempted using the plasmids pMLC27 (*Sr-gpa-3p::GCaMP3::Ss-era-1* 3′ UTR) and pMLC36 (*Sr-gpa-3p::GCaMP6s::Ss-era-1* 3′ UTR). pMLC27 was made by fusing the *Sr-gpa-3* promoter from pAJ09 with *GCaMP3* (Tian *et al.* 2009), and pMLC36 was made by fusing this same promoter with *GCaMP6s* (Chen *et al.* 2013).

### Generation of the *S. stercoralis* stable line

To integrate the *Ss-act-2p::strmScarlet-I* transgene into *S. stercoralis* (Table S1, Exp. 1), 150 free-living females were microinjected with pMLC131 (*Sr-eef-1ap::strhyPBase::Ss-era-1* 3′ UTR; 50 ng/µL) and pRP12 (*Ss-act-2p::strmScarlet-I::Ss-era-1* 3′ UTR; 80 ng/µL) using previously established methods (Castelletto and Hallem 2021) over 2 consecutive days. Five to six days later, a 200-µL packed pellet of F_1_ progeny was recovered. Approximately 100–200 F_1_ iL3s were plated on individual 6-cm plates made of nematode growth medium seeded with a thick lawn of *Escherichia coli* OP50 (Stiernagle 2006) and screened for mScarlet-I fluorescence. A total of 464 transgenic iL3s that expressed the transgene across their entire body wall muscle were picked and placed into 1 mL of BU buffer (Hawdon and Schad 1991). The iL3s were then activated by incubating in Dulbecco's Modified Eagle Medium (DMEM, Gibco, 11995065) at 37°C with 5% CO_2_ for ∼42 h, as described previously (Gang *et al.* 2020). Activated iL3s were washed once with sterile 1X PBS, resuspended in 200 µL of 1X PBS, and inoculated into a single gerbil by oral gavage. Collection of feces, collection of iL3s, and screening for mScarlet-I expression were all done as described above. Subsequent rounds of screening and infection were used to increase the transgenesis rate and maintain the stable line EAH435 *bruIs4*[*Ss-act-2p::strmScarlet-I::Ss-era-1* 3′ UTR] (Table S1, Exp. 1). A second round of microinjection of *S. stercoralis* free-living adults with pMLC131 and pRP12 was done to isolate iL3s that showed mosaic mScarlet-I expression for fluorescence microscopy (Table S1, Exp. 2).

### Generation of *S. ratti* stable lines

To integrate the *Ss-act-2p::strmScarlet-I::Ss-era-1* 3′ UTR transgene into *S. ratti* (Table S1, Exp. 3), 165 free-living females were microinjected with pMLC131 (50 ng/µL) and pRP12 (80 ng/µL), and 35 free-living females were microinjected with the PBase expression vectors pPV402 (*Ss-rps-21p::PBase::Ss-era-1* 3′ UTR; 50 ng/µL) and pRP12 (80 ng/µL) over 3 consecutive days. Five days later, 28 iL3s showing full-body mScarlet-I fluorescence were detected and picked using previously established methods for fluorescence screening of nicotine-paralyzed transgenic iL3s (Gang *et al.* 2017). These iL3s were left in BU buffer overnight and then washed twice and resuspended in sterile 1X PBS the following morning. All iL3s were used to infect a single rat by subcutaneous injection. This attempt yielded the stable line EAH414 *bruIs3*[*Ss-act-2p::strmScarlet-I::Ss-era-1* 3′ UTR], which was maintained by subsequent rounds of screening and infection.

To integrate the *Sr-gcy-23.2p::strYC3.60::Ss-era-1* 3′ UTR transgene into *S. ratti* (Table S1, Exp. 4), 211 free-living females were microinjected with pPV402 (*Ss-rps-21p::PBase::Ss-era-1* 3′ UTR; 50 ng/µL) and pASB63 (*Sr-gcy-23.2p::strYC3.60::Ss-era-1* 3′ UTR; 80 ng/µL; Bryant *et al.* 2022) over 3 consecutive days. A total of 7,700 F_1_ progeny were recovered and screened for the presence of YC3.60 using previously established methods for fluorescence screening of nicotine-paralyzed transgenic iL3s (Gang *et al.* 2017). Fifty-two YC3.60+ F_1_ transgenics were used to infect a rat via subcutaneous injection. As above, subsequent rounds of screening and infection were used to increase the transgenesis rate and maintain the stable line EAH412 *bruIs1*[*Sr-gcy-23.2p::strYC3.60::Ss-era-1* 3′ UTR] (Table S1, Exp. 4).

The same overall methodology was used for the other *S. ratti* stable line attempts, although each attempt differed in specific details such as (1) the transposase expression vector and the transgene–ITR constructs used, as well as the concentration at which each construct was microinjected; (2) the number of P_0_s microinjected; and (3) the number of F_1_ transgenics picked to infect a rat. These details are listed for each stable line attempt in Table S1, Exp. 5-11.

### Fluorescence microscopy

**Fig. 2.**
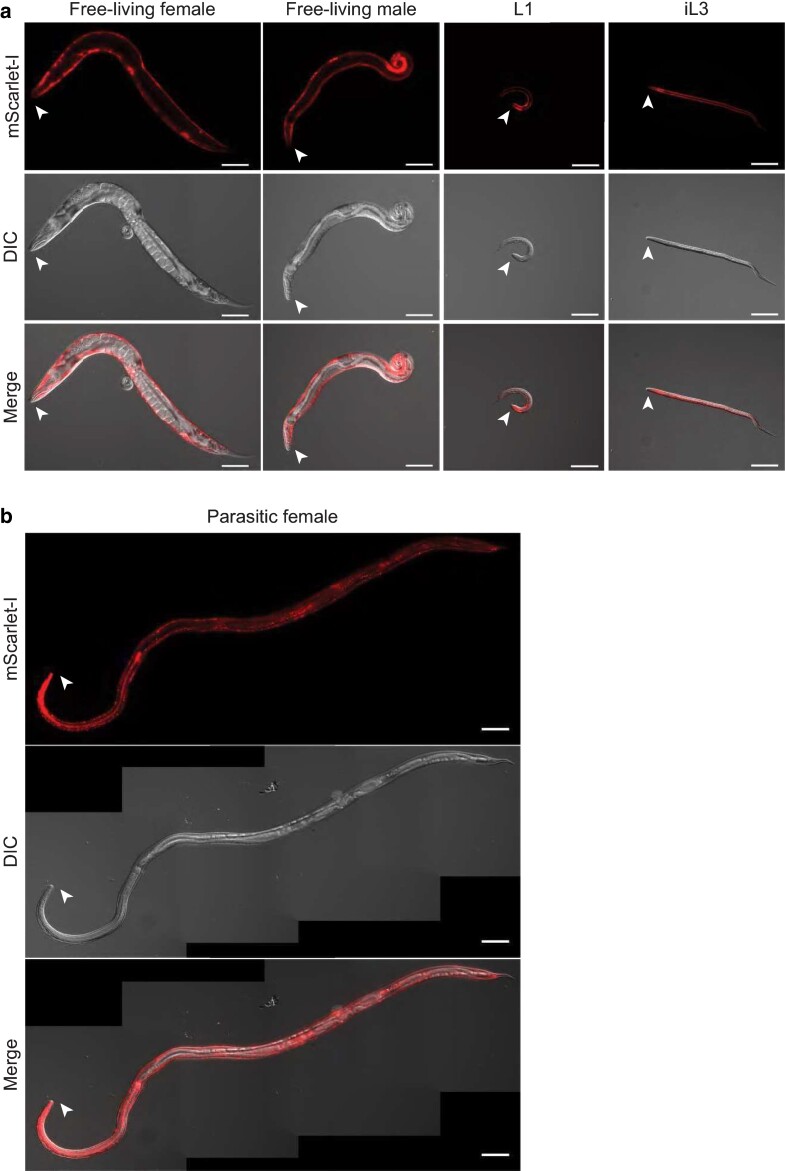
Stable expression of the *Ss-act-2p::strmScarlet-I* transgene in multiple distinct *S. stercoralis* life stages. a) Expression of the hyPBase-integrated *Ss-act-2p::strmScarlet-I* transgene in the body wall muscle of a free-living female, free-living male, post-free-living L1, and iL3. Montage shows mScarlet-I fluorescence (row 1), DIC (row 2), and merged (row 3) images. All worms except the free-living male are oriented with the dorsal side up and ventral side down; the free-living male is oriented with the ventral side up and the dorsal side down. Arrowheads mark the head of the worm. Scale bar = 100 µm. b) Expression of the hyPBase-integrated *Ss-act-2p::strmScarlet-I* transgene in the body wall muscle of a parasitic female isolated from the intestine of a gerbil. Montage shows mScarlet-I fluorescence (row 1), DIC (row 2), and merged (row 3) images. The worm is oriented with dorsal side up and ventral side down. Arrowheads mark the head of the worm. Scale bar = 100 µm.

**Fig. 3.**
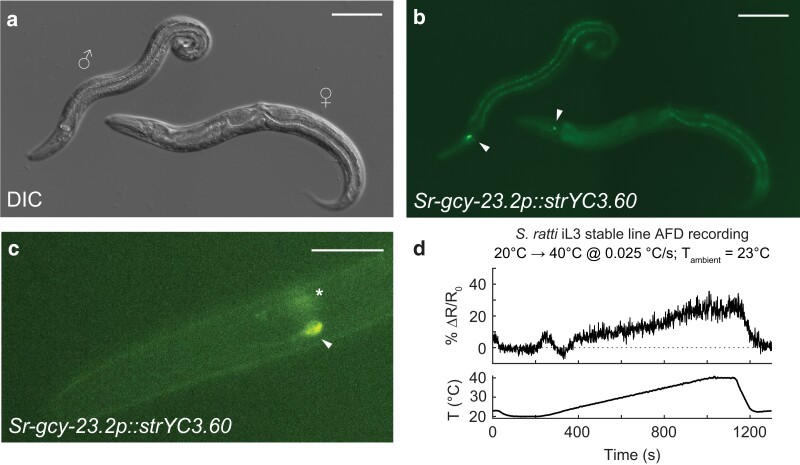
Stable expression of functional transgenes in *S. ratti* AFD neurons using piggyBac transposase. a and b) Expression of the PBase-integrated *Sr-gcy-23.2p::strYC3.60* transgene in the AFD neurons of *S. ratti* free-living adults. a) DIC image of an *S. ratti* free-living male (left) and free-living female (right). b) Expression of *Sr-gcy-23.2p::strYC3.60*. Arrowheads indicate the soma of the *S. ratti* AFD neurons. Scale bar = 100 µm. c) Selective expression of a PBase-integrated *Sr-gcy-23.2p::strYC3.60* transgene in the AFD neurons of an *S. ratti* iL3. Arrowhead indicates an in-focus AFD soma; asterisk indicates the out-of-focus soma of the other AFD neuron. Scale bar = 20 µm. d) Expression of YC3.60 in the AFD neurons of *S. ratti* iL3s from a genome-integrated transgene is sufficient for detecting neuronal responses to thermal stimuli. Trace shows the response of a single *S. ratti* AFD neuron to a 20–40°C warming stimulus. *T*_ambient_ is the temperature at which the worms were cultivated. *R*_0_ (horizontal dotted line) = response at 20°C.

Microscopy of worms was performed using established methods for fluorescence microscopy of nicotine-paralyzed nematodes (Bryant *et al.* 2022). For Fig. 2, transgenic *S. stercoralis* L1s, free-living females, and free-living males were recovered from a 3-day-old fecal–charcoal plate stored at 23°C, and transgenic *S. stercoralis* iL3s were isolated from a 7-day-old fecal–charcoal plate stored at 23°C. The transgenic *S. stercoralis* parasitic adults shown in Fig. 2 and Supplementary Fig. 2 were recovered from the intestine of a single gerbil on day 28 post-infection. Briefly, the gerbil was euthanized, and the intestines were then removed and placed in DMEM preheated to 37°C. The intestines were sliced open and minced, in heated DMEM, using forceps and scissors. Parasitic adults embedded in intestinal tissue were identified using a Leica S6D dissecting microscope and subsequently picked and placed in fresh heated DMEM until imaging. The iL3s with mosaic expression of *Ss-act-2p::strmScarlet-I* shown in Supplementary Fig. 3 were isolated from a 5-day-old fecal–charcoal plate stored at 23°C that contained the progeny of *S. stercoralis* free-living adults that had been microinjected with pMLC131 and pRP12. For Fig. 3, *S. ratti* free-living adults were recovered from 2-day-old fecal–charcoal plates stored at 20°C and screened for YC3.60 expression on a Leica M165 FC fluorescence microscope. All transgenic worms except the *S. stercoralis* parasitic adults were exposed to 10 or 50 mM levamisole in BU saline (Hawdon and Schad 1991), then mounted on slides with 5% Noble agar dissolved in either BU saline or sterile water, coverslipped, and sealed with quick-drying nail polish. Transgenic *S. stercoralis* parasitic adults were exposed to 10 mM levamisole in DMEM and then mounted on slides, as described above.

Epifluorescence and differential interference contrast (DIC) images were taken with either a 20× objective (Plan-Apochromat 20×/0.8 M27; Zeiss) or a 40× oil objective [Plan-Apochromat 40×/1.4 ∞/0.17 Oil DIC (UV) VIS-IR M27; Zeiss] on an inverted Zeiss Axio Observer microscope equipped with a 38 HE filter set for GFP (BP470/40, FT495, BP 525/50), a 63 HE filter set for mScarlet-I (BP572/25, FT590, BP629/62), and a Hamamatsu ORCA-Flash 4.0 camera; fluorescence illumination was provided by Colibri 7 LEDs (LED Module 475 nm). All images were captured using Zeiss ZEN 2 (blue edition) software. Images for the *S. stercoralis* parasitic female, *S. stercoralis* free-living female, and *S. ratti* free-living adults were acquired as tile scans and either exported as stitched images or stitched with the Stitching plugin for ImageJ (Preibisch *et al.* 2009). Image montages for Figs. 2 and 3 and Supplementary Fig. 2 were generated using FIJI (Schindelin *et al.* 2012). The image montage for Supplementary Fig. 3 was generated in Adobe Photoshop 25.5.0.

### Calcium imaging

Microscopy and calcium imaging of EAH412 *bruIs1*[*Sr-gcy-23.2p::strYC3.60::Ss-era-1* 3′ UTR] *S. ratti* iL3s were performed using established methods for thermal stimulation of levamisole-paralyzed transgenic iL3s (Bryant *et al.* 2022). In brief, iL3s were exposed to 100 mM levamisole in BU saline (Hawdon and Schad 1991), then mounted on a slide with 5% Noble agar dissolved in BU, coverslipped, and sealed with quick-drying nail polish. Imaging was performed with a 40× air objective (EC Plan-Neofluar 40×/0.75 ∞/0.17; Zeiss) on an upright Zeiss Axio Imager A2 microscope equipped with a 78 HE ms filter set (BP445/25 + BP510/15, DFT460 + 520 + FT510, BP480/22 + LP530; Zeiss), a Hamamatsu W-View Gemini image splitter with a cyan fluorescent protein (CFP)/yellow fluorescent protein (YFP) dual camera filter set, and a Hamamatsu ORCA-Flash 4.0 camera for simultaneous acquisition of CFP and YFP images. Broad-spectrum fluorescence illumination was provided by an X-Cite Series 210Q lamp. Thermal stimuli were delivered to slide-mounted worms using a custom thermal stimulator, as previously described (Bryant *et al.* 2022). Images were acquired using Zeiss ZEN 2 (blue edition) software; neural responses were extracted and processed as previously described (Bryant *et al.* 2022).

## Results

### Generation of a stable *S. stercoralis* transgenic line using the hyperactive piggyBac transposase hyPBase

We used the piggyBac transposase system to create chromosomal integrants in *S. stercoralis*, as integration using this system was shown previously to prevent transgene silencing in *Strongyloides* spp. (Lok 2012; Shao *et al.* 2012). The wild-type piggyBac transposase (PBase) is an enzyme that excises sequences that are flanked by short ITRs (Fig. 1b; Cary *et al.* 1989; Fraser *et al.* 1995). PBase inserts the excised sequences in a new locus, with high specificity for 5′-TTAA-3′ motifs, duplicating these motifs upon insertion (Fig. 1b; Cary *et al.* 1989).

Our approach for generating integrants in *S. stercoralis* differed from prior attempts in a few key aspects. First, to increase the likelihood of obtaining integrants, we utilized the hyperactive piggyBac transposase (hyPBase), which differs from PBase at 7 amino acids (Supplementary Fig. 1a) and has a higher frequency of integration in mammalian cells (Yusa *et al.* 2011). Second, the sequence encoding hyPBase in the corresponding expression vector was codon-optimized for expression in *Strongyloides* spp. (Supplementary Fig. 1b; Bryant and Hallem 2021), unlike the CDS of PBase that was used previously in *S. stercoralis* (Supplementary Fig. 1c). Third, expression of the *Strongyloides* codon–optimized hyPBase gene, designated *strhyPBase*, was driven by the *Sr-eef-1a* promoter (Fig. 1c), as this promoter is predicted to be highly expressed in the germline and was used successfully to drive the expression of Cas9 for CRISPR/Cas9-mediated mutagenesis (Gang *et al.* 2017).

We microinjected DNA mixtures composed of 2 different plasmids into free-living females to generate *S. stercoralis* integrants (Fig. 1c and d). One plasmid supplied the transgene for integration (hereafter referred to as the transgene–ITR vector), which was a transcriptional reporter for one of the *S. stercoralis* actin genes, *Ss-act-2*, flanked by ITRs (Fig. 1c). The other plasmid was the expression vector for hyPBase (Fig. 1c). In both plasmids, the coding sequences of the genes were fused to the 3′ UTR of *Ss-era-1* because prior studies have shown that inclusion of endogenous regulatory elements in transgenes is required for expression (Li *et al.* 2006; Junio *et al.* 2008). We cultured the free-living females and their progeny on standard fecal–charcoal plates for up to 1 week after microinjection. We then isolated iL3s using a Baermann apparatus and screened for fluorescent worms using a fluorescence dissection microscope (Fig. 1d). While screening, we detected 2 populations of transgenic iL3s: ones with fluorescence signal across their entire body wall muscle (depicted as fully red worms in Fig. 1d), which is consistent with the predicted expression profile of endogenous *Ss-act-2*, and ones with fluorescence signal in patches along the body wall muscle (depicted as partially red worms in Fig. 1d), which were likely mosaic for the transgene (Gang *et al.* 2017). We selected 464 iL3s that showed fluorescence signal throughout their body wall muscle for propagation (Table S1, Exp. 1), because these iL3s were more likely to be integrants (Gang *et al.* 2017). We then activated the selected iL3s in vitro using a standard protocol (Ashton *et al.* 2007; Stoltzfus *et al.* 2012, 2014; Gang *et al.* 2020) and inoculated a single gerbil with activated transgenic iL3s by oral gavage (Fig. 1d). The resulting infection yielded thousands of F_2_/F_3_ larvae, of which approximately 13% were transgenic (Table S1, Exp. 1); this rate of transgenesis is ∼30-fold higher than a prior attempt (Lok 2012), which is likely because of the high integration rate caused by hyPBase. Notably, 85% of the transgenic F_2_/F_3_ larvae showed non-mosaic, full-body expression of the transgene (Table S1, Exp. 1). Thus, the robust infection produced by oral gavage combined with hyPBase-driven chromosomal integration enables the generation and maintenance of stable transgenic *S. stercoralis* lines in laboratory hosts such as gerbils. We have continued to propagate the line for >10 generations.

### *S. stercoralis* stable line shows consistent transgene expression in multiple life stages

After approximately 4 rounds of manual selection for transgenic iL3s followed by passage through gerbils, >99% of the worms recovered from gerbils were transgenic, and all showed bright, full-body expression of the transgene (Fig. 2). We detected bright red fluorescence in the body wall muscle of postparasitic larvae (Fig. 2a), which matches the previously reported expression pattern of the *Ss-act-2* transcriptional reporters (Li *et al.* 2006; Junio *et al.* 2008). The same expression pattern, in the body wall muscle, was also detected in iL3s, free-living adults, and parasitic adults (Fig. 2a and b;Supplementary Fig. 2). Whereas >99% of the worms from the stable line showed full-body expression of the transgene after 4 passages through a gerbil, only ∼20% of the transgenic F_1_ iL3s obtained from microinjection of the same reporter construct showed full-body expression (Table S1, Exp. 2). The remaining transgenic F_1_ iL3s showed variable expression in distinct patches along the body, presumably because of mosaic expression of the transgene from extrachromosomal arrays (Evans 2006); Gang *et al.* 2017; Supplementary Fig. 3). There was seemingly no pattern to the mosaicism, as we detected worms with slivers of red fluorescence in parts of the head, mid-body, and tail (Supplementary Fig. 3). Thus, our data show that hyPBase-driven transgene integration and selective breeding of transgenics with non-mosaic expression profiles lead to a homogeneous stable line, which is consistent with prior findings from *S. ratti* (Shao *et al.* 2012). Moreover, our data demonstrate that robust transgene expression can now be achieved in *S. stercoralis* at any life stage, including post-parasitic life stages.

### Efficient generation of stable *S. ratti* lines using hyPBase

We were also able to efficiently generate stable lines in *S. ratti* using hyPBase. A stable line attempt in *S. ratti* using the hyPBase expression vector and the *Ss-act-2* transcriptional reporter yielded a stable line with scores of transgenic F_2_s and F_3_s—the transgenesis rate was ∼12.6% among the *S. ratti* F_2_/F_3_ generation (Table S1, Exp. 3); this stable line was maintained in the lab for >10 generations. In comparison, the F_2_/F_3_ transgenesis rate of an *S. ratti* yellow cameleon YC3.60 stable line (*Sr-gcy-23.2p::strYC3.60*), which was made using the PBase expression vector, was 30-fold lower than that of the *S. ratti Ss-act-2p::strmScarlet-I* line (Table S1, Exp. 3 and 4), even though almost twice the number of transgenic F_1_s was used to start the infection in the former case. Thus, hyPBase-driven chromosomal integration also improves the efficacy of obtaining and maintaining *S. ratti* stable lines.

Our experiments have also demonstrated that the piggyBac transposase system can be used to integrate transgenes that are expressed in a cell type–specific manner, such as *Sr-gcy-23.2p::strYC3.60* and *Sr-gpa-3p::GFP* (Table S1, Exp. 4 and 8). The *Sr-gcy-23.2p::strYC3.60* transgene was previously shown to label the *S. stercoralis* AFD neurons in F_1_ iL3s that expressed this transgene from extrachromosomal arrays (Bryant *et al.* 2022). Calcium imaging of these transgenic F_1_s during exposure to a thermal gradient showed that the *Ss*-AFD neurons are thermosensory (Bryant *et al.* 2022). By generating the *S. ratti Sr-gcy-23.2p::strYC3.60* stable line, we found that this transgene labels the AFD neurons in other life stages, including the free-living adult stage (Fig. 3a–c). The AFD neurons of an *Sr-gcy-23.2p::strYC3.60* integrant showed dynamic changes in fluorescence when exposed to a thermal gradient (Fig. 3d), showing that the *S. ratti* AFD neurons are also thermosensory and that chromosomal integration preserves transgene function. Thus, hyPBase can likely be used to generate stable lines with various kinds of transgenes, including transcriptional and translational reporters, fluorescent biosensors for neuronal activity, and cassettes for silencing neurons of interest.

## Discussion

**Fig. 4.**
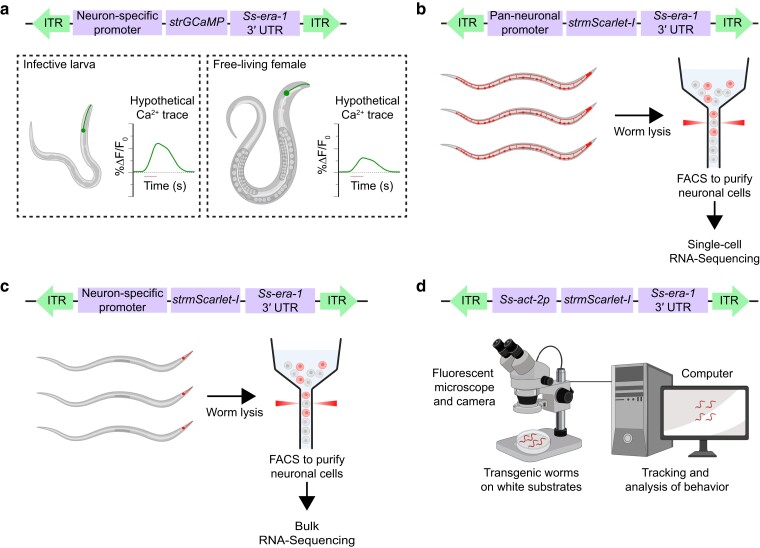
Potential experimental advances using *S. stercoralis* stable lines. a) A stable line expressing a biosensor such as GCaMP under a neuron-specific promoter could be used for comparing the calcium activity of those neurons across life stages, including the iL3 and free-living adult life stages. Hypothetical calcium traces of a neuron of interest are shown for an infective larva and free-living adult that are exposed to a particular stimulus. The change in fluorescence signal over the baseline fluorescence (% Δ*F*/*F*_0_) is plotted on the *y*-axis and time is plotted on the *x*-axis. The line under the *x*-axis indicates the time at which the stimulus is applied. b) A stable line expressing mScarlet-I under a pan-neuronal promoter could be used for isolating neuronal cells from non-neuronal cells by fluorescence-activated cell sorting. Neuronal cells (depicted in color) could then be subjected to single-cell RNA sequencing. c) A stable line expressing mScarlet-I under a neuron-specific promoter could be used for isolating thousands of copies of a particular neuron subtype by fluorescence-activated cell sorting. Neuronal cells (depicted in color) could then be subjected to bulk RNA sequencing. d) The *Ss-act-2p::strmScarlet-I* stable line generated in this paper could be used for assaying worm behavior on substrates where the worms are not visible in white light, such as host skin. Transgenic worms are placed on the substrate, and a fluorescence microscope and camera are used to detect and video-record the worms. Videos are then analyzed post hoc. FACS, fluorescence-activated cell sorting.

Here, we provide an efficient strategy for the generation of stable transgenic lines in both *S. stercoralis* and *S. ratti* that uses the hyperactive piggyBac transposase hyPBase (Fig. 1). Using this strategy, we generated an *S. stercoralis* transgenic line that has been stably maintained in the laboratory for multiple generations (Table S1, Exp. 1). This stable line showed robust transgene expression, in all the predicted cell types, during both intra-host and extra-host life stages (Fig. 2;Supplementary Fig. 2). Additionally, we showed that the piggyBac transposase system can be used to integrate functional transgenes, such as those encoding YC3.60 (Fig. 3); such stable lines could be used for characterizing the activity of specific neurons across multiple life stages (Fig. 4a). We suggest that our approach can be broadly applied to generate stable transgenic lines and thereby bypass issues associated with testing F_1_s, including mosaicism, low transgenesis rates, the need to generate transgenic F_1_s prior to each experimental day, and transgene silencing beyond the F_1_ generation.

We observed that genomic integration driven by hyPBase in *S. ratti* (Exp. 3) produced higher rates of transgenesis in the F_2_/F_3_ generation, as compared with the wild-type piggyBac transposase PBase (Exp. 4). One reason for the difference in transgenesis rates between experiments that used hyPBase and those that used PBase might be that hyPBase improves the efficiency of integration in *Strongyloides* genomes, similar to prior reports on mammalian cells (Yusa *et al.* 2011). Alternatively, the differences in efficiency might be because the CDS of hyPBase in the corresponding expression vector was codon-optimized for expression in *Strongyloides* spp., unlike the PBase CDS (Supplementary Fig. 1b and c). We also cannot exclude the possibilities that the distinct promoters used in the expression vectors for the 2 transposases (Fig. 1c), the inclusion of a synthetic intron in the *strhyPBase* gene in the hyPBase expression vector (Supplementary Fig. 1b), and the different transgenes used for integration may have contributed to differences in the integration efficiencies. We expect our results in *S. ratti* to extend to *S. stercoralis*, since the transgenesis rate we observed using hyPBase in *S. stercoralis* (Exp. 1) was ∼30-fold higher than the transgenesis rate reported in a prior study that used PBase (Lok 2012). Additionally, our findings suggest that there may be a lower limit to the effective concentration of the piggyBac expression vectors for generating chromosomal integrants, since microinjection mixtures that used either the hyPBase expression vector at a concentration of 10 ng/µL (Table S1, Exp. 7) or the PBase expression vector at concentrations lower than 30 ng/µL (Table S1, compare Exp. 6 and 11 with Exp. 9 and 10) yielded no stable lines.

Genomic integration using the piggyBac transposase system offers several advantages over CRISPR/Cas9-mediated integration. First, piggyBac transposase can be used to insert cargo that is up to 207 kilobases (kb) in size (Li *et al.* 2013). In comparison, inserting cargo that is >3 kb by CRISPR/Cas9-mediated genome editing is challenging (Li *et al.* 2014; Ghanta *et al.* 2021). Second, because the piggyBac transposase inserts at 5′-TTAA-3′ motifs (Cary *et al.* 1989), this system can be used to insert transgenes several times into the genome, as compared with CRISPR/Cas9, where the number of insertions is often limited to 2. Additionally, because the *S. stercoralis* genome has an AT content of ∼78% (Hunt *et al.* 2016), 5′-NGG-3′ protospacer adjacent motifs, which are essential for CRISPR/Cas9-mediated genome editing, might not be present at an appropriate location within a gene to allow for endogenous tagging. In such cases, hyPBase-mediated integration of a translational fusion gene, which contains the genomic sequence of the gene of interest fused with a tag (e.g. a 3X-FLAG tag), could be useful for biochemical analyses such as immunoprecipitation followed by mass spectrometry or chromatin immunoprecipitation followed by sequencing.

Before generating a stable transgenic line, some potential disadvantages of the piggyBac transposase system should also be taken into consideration. For instance, because the piggyBac transposase inserts at 5′-TTAA-3′ motifs (Cary *et al.* 1989), which occur in both genic and intergenic regions, transgene integration might cause gene inactivation. As a result, transgenic strains that are generated by this approach might need to be outcrossed, especially if behavioral or developmental phenotypes are observed. Additionally, because the insertion pattern of the piggyBac transposase is semi-random, variable expression of the integrated transgene could occur as a result of either position effects or copy number variation. Indeed, position effects have been observed with transgenic *C. elegans* strains that were generated by random integration (Mello *et al.* 1991).

We have shown that robust *S. stercoralis* infections can be established in gerbils by infecting these animals with activated iL3s via oral gavage. Notably, the infective dose used for this method can be lowered to at least ∼25% of the dose that is typically used for infecting gerbils via subcutaneous injection of iL3s. Additionally, beyond the generation of stable transgenic lines, our approach can be used to generate stable mutant lines for behavioral characterization by oral gavage of activated, mutant F_1_ iL3s, made by CRISPR/Cas9-mediated mutagenesis (Gang *et al.* 2017), into gerbils. In cases where mutation of specific genes might hinder either activation or further development inside of the gerbil, mutant alleles can be maintained in heterozygous worms.

The ever-expanding genetic toolkit of *Strongyloides* now includes an approach for generating stable, heritable *S. stercoralis* lines. Although using transgenic F_1_ animals might be sufficient for certain experiments and could even be advantageous in cases where mosaic analysis of transgene expression is desirable, the ability to generate stable *S. stercoralis* lines will enable several exciting avenues for research in parasite biology. Stable lines expressing functional transgenes that encode YC3.60 and GCaMP can be used for comparing the neuronal responses of iL3s to specific stimuli with those of free-living or parasitic adults to the same stimuli (Fig. 4a). Such studies could pinpoint how behavioral differences among *S. stercoralis* life stages arise from changes in neural circuit function. Additionally, stable lines expressing a fluorescent protein under the control of a promoter of interest could be used for bulk or single-cell RNA sequencing (Fig. 4b and c); a comparison of gene expression signatures in *S. stercoralis* vs *C. elegans* could provide insight into the mechanisms underlying parasite-specific behaviors or developmental programs. Finally, stable lines such as the *Ss-act-2p::strmScarlet-I* line generated in this paper could be used for fluorescence-based visualization of the parasites on substrates such as host skin, where they are undetectable under white light (Fig. 4d). Ultimately, the ability to generate stable transgenic lines in *S. stercoralis* will facilitate mechanistic studies of parasitism and could lead to the identification of genes and pathways to target for preventing or treating infections.

## Supplementary Material

jkae122_Supplementary_Data

## Data Availability

Strains and plasmids are available upon request. The authors affirm that all data necessary for confirming the conclusions of the article are present within the article, figures, and tables. Supplemental material available at G3 online.
