# Supplementary material for: The generation of stable transgenic lines in the human-infective nematode Strongyloides stercoralis
Source: G3 (Bethesda). 2024 Jun 5;14(8):jkae122. doi: 10.1093/g3journal/jkae122 (PMC11304987; doi:10.1093/g3journal/jkae122)
Supplement: jkae122_Supplementary_Data [file jkae122_supplementary_data.pdf]

SUPPLEMENTARY MATERIALS for

The generation of stable transgenic lines in the human-infective nematode *Strongyloides stercoralis*

Ruhi Patel, Astra S. Bryant, Michelle L. Castelletto, Breanna Walsh, Damia Akimori, and Elissa A. Hallem

Correspondence to: ehallem@ucla.edu

This PDF file includes:

Figures S1 to S3

Table S1

Supplemental References

A

```
                  10         20         30         40         50         60         70         80         90        100
Consensus MGSSLDDEHI LSALLQSDDE LVGEDSDSEX SDHVSEDDVQ SDTEEAFIDE VHEVQPTSSG SEILDEQNVI EQPGSSLASN RILTLPQRTI RGKNKHCWST
hyPBase   .......... .......... .........V .......... .......... .......... .......... .......... .......... ..........
PBase     .......... .......... .........I .......... .......... .......... .......... .......... .......... ..........

                 110        120        130        140        150        160        170        180        190        200
Consensus SKXTRRSRVS ALNIVRSQRG PTRMCRNIYD PLLCFKLFFT DEIISEIVKW TNAEISLKRR ESMTXATFRD TNEDEIYAFF GILVMTAVRK DNHMSTDDLF
hyPBase   ..P....... .......... .......... .......... .......... .......... ....S..... .......... .......... ..........
PBase     ..S....... .......... .......... .......... .......... .......... ....G..... .......... .......... ..........

                 210        220        230        240        250        260        270        280        290        300
Consensus DRSLSMVYVS VMSRDRFDFL IRCLRMDDKS IRPTLRENDV FTPVRKIWDL FIHQCIQNYT PGAHLTIDEQ LLGFRGRCPF RXYIPNKPSK YGIKILMMCD
hyPBase   .......... .......... .......... .......... .......... .......... .......... .......... .V........ ..........
PBase     .......... .......... .......... .......... .......... .......... .......... .......... .M........ ..........

                 310        320        330        340        350        360        370        380        390        400
Consensus SGTKYMINGM PYLGRGTQTN GVPLGEYYVK ELSKPVHGSC RNITCDNWFT SIPLAKNLLQ EPYKLTIVGT VRSNKREIPE VLKNSRSRPV GTSMFCFDGP
hyPBase   .......... .......... .......... .......... .......... .......... .......... .......... .......... ..........
PBase     .......... .......... .......... .......... .......... .......... .......... .......... .......... ..........

                 410        420        430        440        450        460        470        480        490        500
Consensus LTLVSYKPKP AKMVYLLSSC DEDASINEST GKPQMVMYYN QTKGGVDTLD QMCSVMTCSR KTNRWPMALL YGMINIACIN SFIIYSHNVS SKGEKVQSRK
hyPBase   .......... .......... .......... .......... .......... .......... .......... .......... .......... ..........
PBase     .......... .......... .......... .......... .......... .......... .......... .......... .......... ..........

                 510        520        530        540        550        560        570        580        590
Consensus KFMRNLYMXL TSSFMRKRLE APTLKRYLRD NISNILPXEV PGTSDDSTEE PVMKKRTYCT YCPSKIRRKA XASCKKCKKV ICREHNIDMC QSCF*
hyPBase   ........G. .......... .......... .......K.. .......... .......... .......... S......... .......... .....
PBase     ........S. .......... .......... .......N.. .......... .......... .......... N......... .......... .....
```

B ***strhyPBase***

```
ATGGGATCTT CTCTTGATGA TGAACATATT CTTTCTGCTC TTCTTCAATC TGATGATGAA CTTGTTGGAG AAGATTCTGA TTCTGAAGTT TCTGATCATG
TTTCTGAAGA TGATGTTCAA TCTGATACTG AAGAAGCCTT CATTGATGAA GTTCATGAAG TTCAACCAAC TTCTTCTGGA TCTGAAATTC TTGATGAACA
AAATGTTATT GAACAACCAG GATCTTCTCT TGCTTCTAAT CGTATTCTTA CTCTTCCACA ACGTACTATT CGTGGAAAAA ATAAACATTG TTGGTCTACT
TCTAAACCAA CTCGTCGTTC TCGTGTTTCT GCTCTTAATA TTGTTCGTTC TCAACGTGGA CCAACTCGTA TGTGTCGTAA TATTTATGAT CCACTTCTTT
GTTTCAAACT TTTCTTCACT GATGAAATTA TTTCTGAAAT TGTTAAATGG ACTAATGCTG AAATTTCTCT TAAACGTCGT GAATCTATGA CTTCTGCTAC
TTTCCGTGAT ACTAATGAAG GTAAGTTTAA ACATATATAT ACTAACTAAC CCTGATTATT TAAATTTTCA GATGAAATTT ATGCTTTCTT CGGTATTCTT
GTTATGACTG CTGTTCGTAA AGATAATCAC ATGTCTACTG ATGATCTTTT TGATCGTTCT CTTTCTATGG TTTATGTTTC TGTTATGTCT CGTGATCGTT
TCGATTTCCT TATTCGTTGT CTTCGTATGG ATGATAAATC TATTCGTCCA ACTCTTCGTG AAAATGATGT TTTCACTCCA GTTCGTAAAA TTTGGGATCT
TTTCATTCAT CAATGTATTC AAAATTATAC TCCAGGTGCT CATCTTACTA TTGATGAACA ACTTCTTGGA TTCCGTGGAC GTTGTCCATT CCGTGTTTAT
ATTCCAAATA AACCATCTAA ATATGGAATT AAAATTCTTA TGATGTGTGA TTCTGGAACT AAATATATGA TTAATGGAAT GCCATATCTT GGACGTGGAA
CTCAAACTAA TGGAGTTCCA CTTGGAGAAT ATTATGTTAA AGAACTTTCT AAACCAGTTC ATGGATCTTG TCGTAATATT ACTTGTGATA ATTGGTTCAC
TTCTATTCCA CTTGCTAAAA ATCTTCTTCA AGAACCATAT AAACTTACTA TTGTTGGAAC TGTTCGTTCT AATAAACGTG AAATTCCAGA AGTTCTTAAA
AATTCTCGTT CTCGTCCAGT TGGAACTTCT ATGTTCTGTT TCGATGGACC ACTTACTCTT GTTTCATATA AACCAAAACC AGCTAAAATG GTTTATCTTC
TTTCTTCTTG TGATGAAGAT GCTTCTATTA ATGAATCTAC TGGAAAACCA CAAATGGTTA TGTACTACAA TCAAACTAAA GGAGGAGTTG ATACTCTTGA
TCAAATGTGT TCTGTTATGA CTTGTTCTCG TAAAACTAAT CGTTGGCCAA TGGCTCTTCT TTATGGAATG ATTAATATTG CTTGTATTAA TTCTTTCATT
ATTTATTCTC ATAATGTTTC TTCTAAAGGA GAAAAAGTTC AATCTCGTAA AAAATTCATG CGTAATCTTT ATATGGGACT TACTTCTTCT TTCATGCGTA
AACGTCTTGA AGCTCCAACT CTTAAACGTT ATCTTCGTGA TAATATTTCT AATATTCTTC CAAAAGAAGT TCCAGGAACT TCTGATGATT CTACTGAAGA
ACCAGTTATG AAAAAACGTA CTTATTGTAC TTATTGTCCA TCTAAAATTC GTCGTAAAGC CTCTGCTTCT TGTAAAAAAT GTAAAAAAGT TATTTGTCGT
GAACATAATA TTGATATGTG TCAATCTTGT TTCTAA
```

■ CDS ■ Syntron ■ Codons that encode amino acids specific to hyPBase

C ***PBase***

```
ATGGGATGTT CTTTAGACGA TGAGCATATC CTCTCTGCTC TTCTGCAAAG CGATGACGAG CTTGTTGGTG AGGATTCTGA CAGTGAAATA TCAGATCACG
TAAGTGAAGA TGACGTCCAG AGCGATACAG AAGAAGCGTT TATAGATGAG GTACATGAAG TGCAGCCAAC GTCAAGCGGT AGTGAAATAT TAGACGAACA
AAATGTTATT GAACAACCAG GTTCTTCATT GGCTTCTAAC AGAATCTTGA CCTTGCCACA GAGGACTATT AGAGGTAAGA ATAAACATTG TTGGTCAACT
TCAAAGTCCA CGAGGCGTAG CCGAGTCTCT GCACTGAACA TTGTCAGATC TCAAAGAGGT CCGACGCGTA TGTGCCGCAA TATATATGAC CCACTTTTAT
GCTTCAAACT ATTTTTTACT GATGAGATAA TTTCGGAAAT TGTAAAATGG ACAAATGCTG AGATATCATT GAAACGTCGG GAATCTATGA CAGGTGCTAC
ATTTCGTGAC ACGAATGAAG ATGAAATCTA TGCTTTCTTT GGTATTCTGG TAATGACAGC AGTGAGAAAA GATAACCACA TGTCCACAGA TGACCTCTTT
GATCGATCTT TGTCAATGGT GTACGTCTCT GTAATGAGTC GTGATCGTTT TGATTTTTTG ATACGATGTC TTAGAATGGA TGACAAAAGT ATACGGCCCA
CACTTCGAGA AAACGATGTA TTTACTCCTG TTAGAAAAAT ATGGGATCTC TTTATCCATC AGTGCATACA AAATTACACT CCAGGGGCTC ATTTGACCAT
AGATGAACAG TTACTTGGTT TTAGAGGACG GTGTCCGTTT AGGATGTATA TCCCAAACAA GCCAAGTAAG TATGGAATAA AAATCCTCAT GATGTGTGAC
AGTGGTACGA AGTATATGAT AAATGGAATG CCTTATTTGG GAAGAGGAAC ACAGACCAAC GGAGTACCAC TCGGTGAATA CTACGTGAAG GAGTTATCAA
AGCCTGTGCA CGGTAGTTGT CGTAATATTA CGTGTGACAA TTGGTTCACC TCAATCCCTT TGGCAAAAAA CTTACTACAA GAACCGTATA AGTTAACCAT
TGTGGGAACC GTGCGATCAA ACAAACGCGA GATACCGGAA GTACTGAAAA ACAGTCGCTC CAGGCCAGTG GGAACATCGA TGTTTTGTTT TGACGGACCC
CTTACTCTCG TCTCATATAA ACCGAAGCCA GCTAAGATGG TATACTTATT ATCATCTTGT GATGAGGATG CTTCTATCAA CGAAAGTACC GGTAAACCGC
AAATGGTTAT GTATTATAAT CAAACTAAAG GCGGAGTGGA CACGCTAGAC CAAATGTGTT CTGTGATGAC CTGCAGTAGG AAGACGAATA GGTGGCCTAT
GGCATTATTG TACGGAATGA TAAACATTGC CTGCATAAAT TCTTTTATTA TATACAGCCA TAATGTCAGT AGCAAGGGAG AAAAGGTTCA AAGTCGCAAA
AAATTTATGA GAAACCTTTA CATGAGCCTG ACGTCATCGT TTATGCGTAA GCGTTTAGAA GCTCCTACTT TGAAGAGATA TTTGCGCGAT AATATCTCTA
ATATTTTGCC AAATGAAGTG CCTGGTACAT CAGATGACAG TACTGAAGAG CCAGTAATGA AAAAACGTAC TTACTGTACT TACTGCCCCT CTAAAATAAG
GCGAAAGGCA AATGCATCGT GCAAAAAATG CAAAAAAGTT ATTTGTCGAG AGCATAATAT TGATATGTGC CAAAGTTGTT TC
```

■ CDS

Figure S1. The amino acid and DNA sequences of the hyPBase and PBase that were used in the corresponding expression vectors to drive chromosomal integration. A. The amino acid sequence of hyPBase differs from that of PBase at 7 amino acids. The consensus sequence from the alignment of hyPBase (YUSA *et al.* 2011) and PBase (FRASER *et al.* 1995) is shown. Within the consensus sequence, amino acids that are different in hyPBase and PBase (FRASER *et al.* 1995; SHAO *et al.* 2012) are labeled "X " The amino acids at these positions are listed for each transposase. **B.** The *strhyPBase* gene was codon-optimized for expression in the *Strongyloides* genome. The gene encoding hyPBase in the expression vector used for integration is shown. The CDS is in black, a synthetic intron is in purple, and the codons encoding the amino acids specific to hyPBase are highlighted in turquoise. **C.** The PBase gene is not codon-optimized for expression in the *Strongyloides* genome. The gene encoding PBase in the expression vector used for integration is shown (SHAO *et al.* 2012). The CDS is labelled black. No synthetic intron was included in this vector.

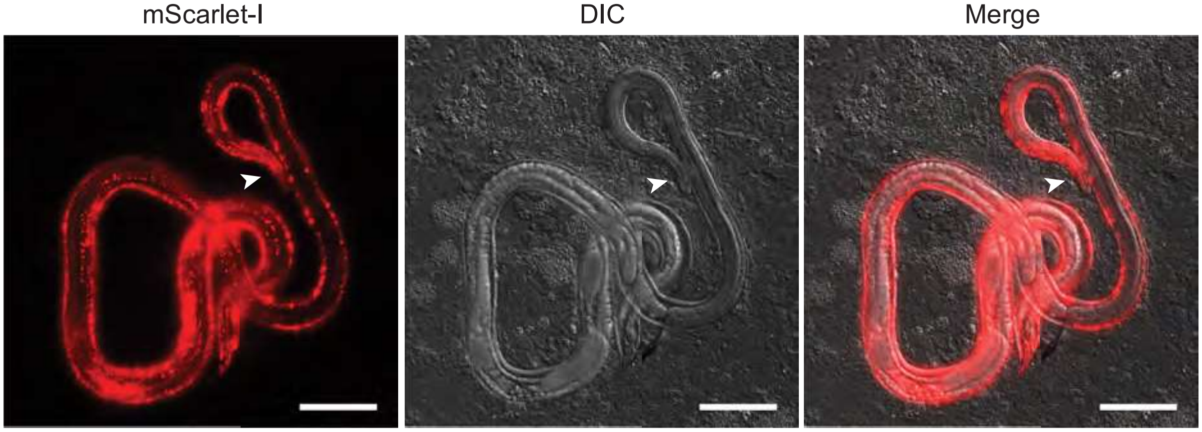

Figure S2. A parasitic female embedded in host intestinal tissue shows bright expression of the hyPBase-integrated *Ss-act-2p::strmScarlet-I* transgene. Expression of the hyPBase-integrated *Ss-act-2p::strmScarlet-I* transgene in the body wall muscle of a parasitic female trapped in gerbil intestinal tissue. Montage shows mScarlet-I fluorescence, DIC, and merged images. The debris outside of the parasitic adult, which is visible in the DIC and merged images, is likely deteriorated intestinal tissue. Arrowheads mark the head of the worm. Scale bar = 100 µm.

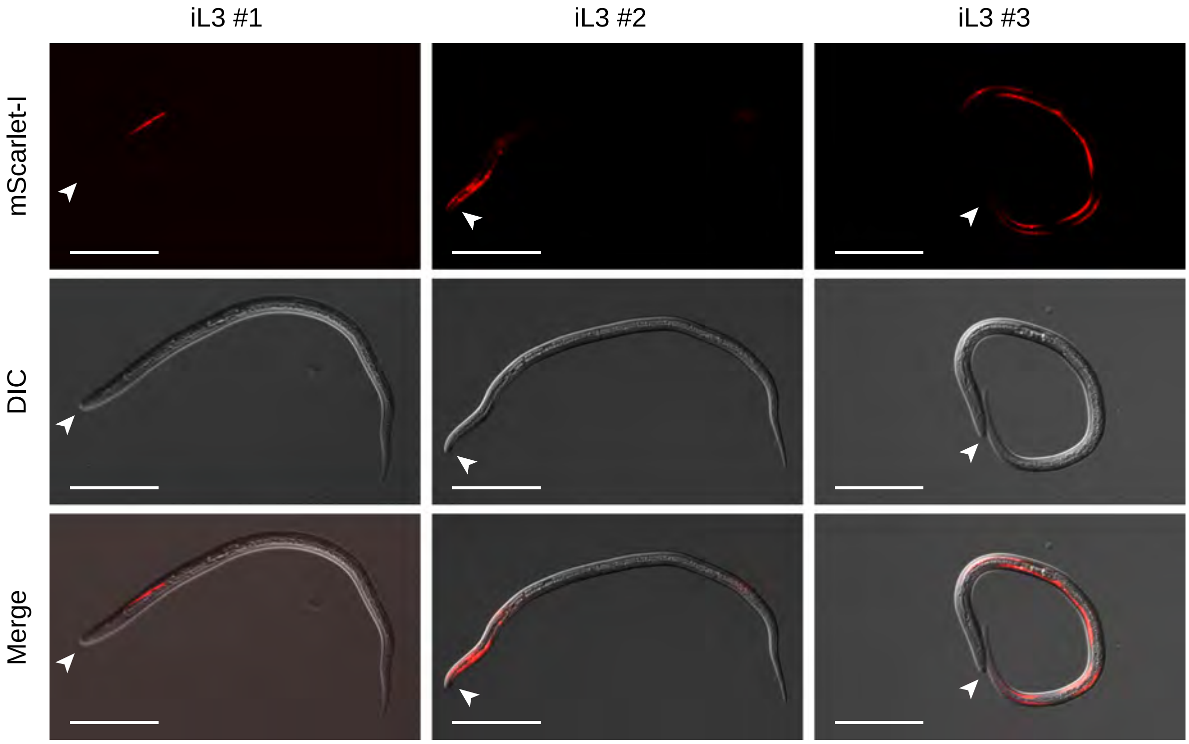

Figure S3. Mosaic expression of *Ss-act-2p::strmScarlet-I* from an extrachromosomal array. Variable expression of *Ss-act-2p::strmScarlet-I* in three distinct F_1 iL3s, labeled #1, #2 and #3, that likely express the transgene from extrachromosomal arrays. Montage shows mScarlet-I fluorescence, DIC, and merged images. The worms are oriented with dorsal side down and ventral side up. Arrowheads mark the head of the worm. Scale bar = 100 µm.

Table S1. Higher rates of integration are achieved with hyPBase than PBase. The table lists all microinjections done in this study. For each experiment, details listed include the species tested, the experimental goals, the constructs used, the concentrations at which each construct was microinjected, the number of free-living females injected, the percent transgenesis in the F_1 generation, the number of F_1 transgenics used to infect a single animal, whether a stable line was established, and the percent transgenesis in the F_2/F_3 generation. In Exp. 3, some animals were injected with hyPBase and some with PBase because this was one of our first experiments with hyPBase; since there was no prior evidence that hyPBase would drive integration in *Strongyloides* spp., we injected a subset of animals with PBase to increase the likelihood of obtaining integrants. n.d. = not determined. *There were no ITRs in the backbone of the *Ss-act-2p::mRFPmars* construct; it was used only as a co-injection marker. **The stable transgenic line that was established showed expression of *Ss-act-2p::mRFPmars* but not *Ss-gpa-3p::GCaMP6s* (Exp. 9) or *Ss-gpa-3::GCaMP3* (Exp. 10), even though all three plasmids had ITRs.

Exp #	Species	Experimental goal	Constructs injected	Conc. (ng/µL)	# P_0s injected	% transgenesis in F_1 generation	# F_1 transgenics picked	Stable line established (Y/N)	% transgenesis in F_2/F_3 generation
1	*S. stercoralis*	Expression of mScarlet-I in body wall muscle	**hyPBase vector**	50	150	n.d.	464	Y	12.8 (85% full-body; 15% patchy)
			Ss-act-2p::strmScarlet-I	80					
2	*S. stercoralis*	Expression of mScarlet-I in body wall muscle	**hyPBase vector**	50	20	11.25 (21% full-body; 79% patchy)	-	-	-
			Ss-act-2p::strmScarlet-I	80					
3	*S. ratti*	Expression of mScarlet-I in body wall muscle	**hyPBase vector**	50	165	n.d.	28	Y	12.6
			Ss-act-2p::strmScarlet-I	80					
			**PBase vector**	50	35				
			Ss-act-2p::strmScarlet-I	80					
4	*S. ratti*	Expression of yellow cameleon in AFD neurons	**PBase vector**	50	211	0.68	52	Y	0.41
			Sr-gcy-23.2p::strYC3.60	80					
5	*S. ratti*	Expression of yellow cameleon in AFD neurons	**PBase vector**	35	144	n.d.	35	N	0.00
			Sr-gcy-23.2p::strYC3.60	80					
6	*S. ratti*	Expression of yellow cameleon in AFD neurons	**PBase vector**	10	243	2.12	98	N	0.00
			Sr-gcy-23.2p::strYC3.60	80					
7	*S. ratti*	Expression of yellow cameleon in AFD neurons	**hyPBase vector**	10	239	2.57	15	N	0.00
			Sr-gcy-23.2p::strYC3.60	80					
8	*S. ratti*	Expression of mRFPmars in body wall muscle and GFP in head neurons	**PBase vector**	35	53	2.20	16	Y	n.d.
			Sr-gpa-3p::GFP	50					
			*Ss-act-2p::mRFPmars**	20					
9	*S. ratti*	Expression of mRFPmars in body wall muscle and GCaMP6s in head neurons	**PBase vector**	30	126	0.40	21	Y**	n.d.
			Ss-gpa-3p::GCaMP6s	50					
			Ss-act-2p::mRFPmars	20					
10	*S. ratti*	Expression of mRFPmars in body wall muscle and GCaMP3 in head neurons	**PBase vector**	30	150	n.d.	12	Y**	n.d.
			Ss-gpa-3p::GCaMP3	50					
			Ss-act-2p::mRFPmars	20					
11	*S. ratti*	Expression of mRFPmars in body wall muscle and GCaMP3 in head neurons	**PBase vector**	20	102	n.d.	21	N	0.00
			Ss-gpa-3p::GCaMP3	65					
			Ss-act-2p::mRFPmars	12					

SUPPLEMENTAL REFERENCES

Fraser, M.J., L. Cary, K. Boonvisudhi and H.G. Wang, 1995 Assay for movement of Lepidopteran transposon IFP2 in insect cells using a baculovirus genome as a target DNA. Virology 211**:** 397-407.

Shao, H., X. Li, T.J. Nolan, H.C. Massey, Jr., E.J. Pearce *et al.*, 2012 Transposon-mediated chromosomal integration of transgenes in the parasitic nematode *Strongyloides ratti* and establishment of stable transgenic lines. PLoS Pathog 8**:** e1002871.

Yusa, K., L. Zhou, M.A. Li, A. Bradley and N.L. Craig, 2011 A hyperactive piggyBac transposase for mammalian applications. Proc Natl Acad Sci USA 108**:** 1531-1536.
